# Co‐Design of the MAGNET Intervention to Enhance Medicines Self‐Management in Community‐Dwelling People With Dementia and Their Family Carers

**DOI:** 10.1111/hex.70760

**Published:** 2026-10-15

**Authors:** Catherine Powell, Lubena Mirza, Mohammed Akhlak Rauf, Justine Tomlinson, Zoe Edwards, Rozeena Khan, Jan Speechley, John L. O'Dwyer, Catherine Quinn, Beth Fylan

**Affiliations:** ^1^ School of Pharmacy, Optometry and Medical Sciences University of Bradford Bradford West Yorkshire UK; ^2^ Wolfson Centre for Applied Health Research Bradford West Yorkshire UK; ^3^ NIHR Yorkshire and Humber Patient Safety Translational Research Centre Bradford Institute for Health Research Bradford West Yorkshire UK; ^4^ Patient & Public Involvement Group Lead Meri Yaadian Bradford West Yorkshire UK; ^5^ Affinity Care PCN Bradford West Yorkshire UK; ^6^ MAGNET PPI group University of Bradford Bradford West Yorkshire UK; ^7^ Academic Unit of Health Economics, Leeds Institute of Health Sciences University of Leeds, Worsley Building Leeds West Yorkshire UK; ^8^ Department of Applied Dementia Studies University of Bradford Bradford West Yorkshire UK

**Keywords:** coproduction, dementia, medicines, methods, PPI, resilience, self‐management

## Abstract

**Background:**

With appropriate support people living with mild‐moderate dementia and family carers can self‐manage their medicines. However, existing interventions often focus on enhancing adherence to a medication regimen rather than supporting people with other areas, such as monitoring side effects or checking for errors. A person‐centred psychosocial intervention, empowering community‐dwelling people living with mild‐moderate dementia and family carers to self‐manage their medicines, is warranted. The method of developing such an intervention needs to reflect this person‐centred approach.

**Objective:**

To co‐design a psychosocial intervention to support medicines self‐management for community‐based people living with mild‐moderate dementia and family carers.

**Design:**

An Experience‐Based‐Co‐Design (EBCD) approach was applied to develop the ‘Enhancing medicines self‐management in community‐dwelling people living with dementia and family carers (MAGNET)’ intervention. The EBCD method involves intervention design through service users identifying problems they face and co‐designing solutions with service providers. A film about experiences of medicines self‐management was shown to people with dementia, family carers, and professionals; followed by a priority setting workshop, six intervention co‐design workshops and a celebration event. Feedback was collated. A complex psychosocial healthcare intervention was developed.

**Setting and Stakeholders:**

Community‐dwelling people living with dementia and family carers, dementia care professionals, an expert facilitator, members of the MAGNET patient public involvement (PPI) group and members of the MAGNET research team.

**Results:**

The resulting co‐designed intervention focused on five key priority areas: ‘how can living with dementia affect how you organise medicines’, ‘knowing my medicines’, ‘who can help’, ‘making it right for you’ and ‘planning for the future’. A guide, video and reminder postcard for the person, and training and an implementation guide for professionals were created, alongside a logic model. Stakeholders reported positive experiences in developing the intervention, including giving back to the community, and the opportunity learn from and gain support from others.

**Conclusion:**

The process of co‐designing the intervention had an immediate important social impact. Further work is underway to feasibility test the intervention.

**Patient or Public Contribution:**

Patient public involvement members have had a key role in co‐designing the intervention, participating in and co‐leading workshops and reviewing iterations.

## 1 Introduction

Creating safer medicine self‐management systems for people living with dementia and their family carers by co‐creating usable solutions is a critical international issue. Over 55 million people worldwide are living with dementia [1]. In the UK alone, there are over 982,000 people living with dementia [2]. The cognitive decline associated with dementia can make medicines management more challenging; for example, people may forget to take their medications [3], and evidence suggests as many of 19% of people living with dementia in the community experience adverse drug events [4]. The management of medicines across multiple health, social and voluntary sector organisations can be enhanced through patients' actions to manage their medicines [5, 6]. Yet, there is insufficient community support from professionals for a safe medicines management system for community dwelling people with dementia. People may therefore be left to develop their own strategies unsupported, which could introduce risks [7].

Self‐management interventions offer a way of supporting people, through empowering them to self‐manage their condition according to their own psychosocial lived experience, through setting goals, enhancing knowledge and skills, and monitoring and evaluating [8]. Self‐management interventions have been shown to enhance quality of life, self‐efficacy, knowledge and skills to manage illness, and reduce stress for people with dementia [9]. Such interventions are most relevant to those in the early stages given cognitive decline [10]. Therefore, a self‐management approach could be applied to medicines safety.

Self‐management of medicines has been defined as ‘the extent to which a patient takes medication as prescribed, including not only the correct dose, frequency, and spacing but also its continued, safe use over time’ [11]. A more person‐centred approach to medicines self‐management is needed, that encompasses more than adherence [12, 13]. Our systematic review of interventions for self‐management of medicines for community‐dwelling people with dementia and mild cognitive impairment and their family carers identified that existing interventions more often focused on enhancing adherence, as outlined in the definition above. A more holistic, person‐centred view of medicines self‐management could consider patient's skills, self‐efficacy and resilience capabilities in checking for errors, seeking support, adherence, knowledge, supply management and monitoring effects [14].

Introducing theory that aligns with a person‐centred approach could enhance a medicines self‐management intervention. Theoretically underpinned interventions are more likely to be successful in terms of sustainability, effectiveness and scalability [15]. Our systematic review identified there were limited interventions which had a theoretical basis. Theories were social cognitive and self‐efficacy theories, by which individuals hold a belief that they can complete an action, have been underpinning theories of a limited number of self‐management interventions [16, 17]. We identified that a resilient healthcare (Safety II) approach which shows how safe outcomes can be reached depending on the flexibility and adaptability of healthcare systems, could be a critical underpinning theory [18].

As well as having an appropriate theoretical underpinning, the method of developing an intervention should also take a person‐centred approach. Experience‐Based‐Co‐Design (EBCD) is a participatory method, where patients, public and professionals involved in a service are brought together to co‐design services [19]. Whilst this method is usually conducted in single organisations for service improvement purposes, EBCD has been applied in several ways [20], and it has been implemented across multiple sites and for research purposes [21]. An EBCD approach to medication management post‐hospitalisation for people living with dementia was conducted in Australia. This led to the co‐design of a simplified medication management guide for people living with dementia and a carer‐focused guide. Stakeholders co‐developed topics around informed consent, shared decision making, and medications affecting cognition [22]. A theoretically informed EBCD intervention supporting medicines self‐management for people with mild‐moderate dementia with family carers is needed. We therefore sought to follow an EBCD process for a psychosocial intervention with a focus on medicines self‐management for people with mild‐moderate dementia and family carers living at home in the UK.

## 2 Materials and Methods

The key aim was to co‐design an intervention for people living with mild‐moderate dementia, with and without family carers, to safely self‐manage their medicines.

The objectives were to:

- co‐design an intervention with people living with mild‐moderate dementia, family carers and health and social care professionals to support medicine self‐management strategies.
- develop a theory of change to support the co‐designed intervention.

### 2.1 Preliminary Work

We followed an evidence‐based version of EBCD that had been adapted from a service improvement tool to use for multisite research projects developing patient‐centered interventions [21, 23], based on an integration of EBCD and Medical Research Council (MRC) guidelines for the development of complex research interventions [24]. There were three phases in the project. In this paper, we report the findings of phase two. Phase two was informed by project phase one, referred to throughout this paper as preliminary work. In phase one, we explored how people with mild‐moderate dementia with and without their family self‐managed their medicines. Interviews with community dwelling people living with mild‐moderate dementia, their family carers and professionals were carried out. Extracts from some of the interviews were used to create a film for the project phase two. The film involves people with mild‐moderate dementia with and without carers describing experiences of self‐managing their medicines. In addition, the team had previously conducted a systematic review of the literature on interventions to enhance medicines self‐management for people living with dementia and mild cognitive impairment [14].

These findings provided the research team with an early understanding of what the intervention might include, and these preliminary ideas were shared with a new group of stakeholders during the project phase two EBCD process through presentation and discussion. Key theoretical underpinnings for the psychosocial intervention included social cognitive and self‐efficacy theory [25, 26], and resilient healthcare theory [18]. These theories underpinned our discussion in the meetings. We discussed, enhancing people's resilience and self‐efficacy for self‐managing their medicines through checking for errors, seeking support, adherence, knowledge, supply management and monitoring effects, as a core focus of the intervention.

Ethical approval was granted by the Research Ethics Committee and UK Health Research Authority REC: 23/PR/0180/IRAS: 320482. This project phase 2 (co‐designing the intervention of the MAGNET project) was a development activity, with the stakeholders involved as collaborators. Therefore, informed consent was not required. Personal information about people was not captured. Discussions were not recorded nor subjected to thematic analysis. For project phase one, research participants all gave informed consent.

## 3 Engaging With Stakeholders

We invited people with dementia, family carers and professionals to collaborate with the research team to co‐design the intervention. Inclusion criteria for these stakeholders were:

• Living with mild‐moderate dementia (as indicated by a Mini‐Mental State Examination [27] score between 24 and 10 or a clinical diagnosis of mild‐moderate dementia), from diverse backgrounds. Using two or more medicines. Living at home. Living alone or living with family carers.

• Relatives, friends or neighbours identified as involved in supporting the person with their medicines. Caring for the person with mild‐moderate dementia (as indicated by a Mini‐Mental State Examination [27] score between 24 and 10, or a clinical diagnosis of mild‐moderate dementia), using two or more medicines, and living at home. May be living within or outside the household of the person with dementia.

• Health, social care or voluntary sector professionals who support people with dementia and family carers or researchers with relevant health, voluntary or social care background.

In addition to inviting new stakeholders, the existing MAGNET patient public involvement (PPI) group were invited to collaborate with us to co‐design of the intervention. Our PPI group lead is a co‐investigator on the project. The group consists of five people including a person living with dementia and former family carers of people with dementia. The group has advised and conducted research activities throughout all the project phases. They were involved in initial project design. GRIPP2 long form reporting guidelines and reporting template (Supporting Information S1: Additional File 1) have been followed [28].

We aimed to carry out EBCD in three different urban and rural geographical areas in Northern England. People living with dementia, family carers, and health and social care professionals identified from previously conducted phase one interviews were invited, with additional stakeholders identified and invited via networks. We aimed for a mix of people with dementia, family carers and professionals from each geographical area. Face‐to‐face or virtual workshops, at a maximum 1.5 h, were offered to reduce burden and facilitate meeting attendance. Where possible, meetings were held in familiar community settings or held at the health research institution. Time was made for introductions, scene setting and breaks. Staff were available to support individuals at each of the meetings. Meetings were held between 16th January 2024 and 28th June 2024. Stakeholders and the PPI group were paid for their time and any travel expenses.

An outline of the EBCD process appears in Table 1.

**Table 1:** Planned process to co‐design the intervention.

Phase	Stakeholders and activity	Intended outcome	Activities post	Lead facilitator
Preliminary work (Project phase 1)	Systematic literature review and qualitative interviews with stakeholders	Thematic analysis: Evidence of existing medicines self‐management interventions, and theoretical underpinnings	Create film and evidence summary	Research team
1a. Priority setting	Group meetings in 3 different geographical areas. 6 People with dementia and family carers per area, Discussing evidence from the first part of our project (a film, data analysis and systematic literature review)	Meeting to identify a long list of priorities for change and improvement	Time to read through meeting list and notes	MAGNET project team representative
1b. Priority setting	Group meetings in 3 different geographical areas. 3 health and social care professionals per area, Discussing evidence from the first part of our project (a film, data analysis and systematic literature review)	Meeting to identify a long list of priorities for change and improvement	Time to read through meeting list and notes	MAGNET project team representative
1c. Priority setting	Joint feedback event with all 27 stakeholders from the 3 geographical areas	Identify 3 key priorities to be taken to next phase	Preparing any materials for the next phase	MAGNET project team representative, PPI member and external facilitator
2a. Co‐designing the intervention	Co‐designing the intervention and ideas about facilitating. 2 co‐design workshops in each of the 3 geographical areas. Each area 6 people with dementia and family, and 3 health and social care professionals	Co‐designed solutions for the key priorities identified in phase 2	Create the physical intervention materials	MAGNET project team representative, PPI member and external facilitator
2b. Co‐designing the intervention	Celebration event with all 27 stakeholders	Celebration of achievement, final refining of co‐designed solution and feedback on the EBCD process	Final intervention materials developed, and feedback reported on	MAGNET project team representative, PPI member and external facilitator

In ‘Priority‐Setting phases 1a and1b’, the findings of the systematic review were shared with stakeholders including the person‐centred definition of medicines self‐management, supporting people, through empowering them to self‐manage their condition through setting goals, enhancing knowledge and skills, and monitoring and evaluating, underpinned by social cognitive and self‐efficacy theories [16, 17], and resilience abilities: ability to respond, such as being able to respond to changes; ability to monitor, awareness of what to look out for; ability to learn, awareness of what has happened and being able to learn from this; and ability to anticipate, such as knowing what to expect [14, 18]. The film of people with mild‐moderate dementia with and without carers describing experiences of self‐managing medicines was shown. The film was intended to generate thought and discussion to help identify the key priorities, what people with dementia, family carers and professionals felt was really needed, to enhance medicines self‐management for people with mild‐moderate dementia through a psychosocial intervention. Following EBCD methodology, people living with dementia and family carers were in separate groups to professionals in the initial ‘1a and 1b priority‐setting’ meetings. A joint priority‐setting meeting was held following this, with all stakeholders invited to attend. The purpose was to review and select the most important priorities from the previously identified longer list of priorities from phases 1a meetings with people with dementia and carers, and 1b meetings with professionals. The joint meeting was led by a facilitator from a visual communication agency, with the aim of communicating creatively and accessibly with stakeholders.

### 3.1 Co‐Design Meetings

Phase ‘2a Co‐designing the intervention’ meetings were held in the 3 separate geographical areas with a mix of people with dementia, family carers and health and care professionals. Some meetings were led by the external facilitator from a visual communication agency and some by the PPI lead. The process involved writing content for the intervention prototypes and deciding on the design of the prototypes. In ‘Phase 2b Co‐designing the intervention’, the celebration event, a sample of the prototypes were reviewed, and feedback on the EBCD process itself was provided verbally and on flip chart paper in mixed groups of people with dementia, family carers, professionals. PPI group members were involved as facilitators and/or stakeholders in different phases as indicated in Table 1. The intervention and implementation materials were refined, alongside the development of a logic model. The model aims to explain how the intervention could meet outcomes to enhance medicines self‐management for people with dementia and family carers.

## 4 Results

Stakeholders at each of the meetings are summarised in Table 1. 1a and 1b meetings were held separately. In 1c, priorities from the earlier meetings were discussed. In 2a there were three groups (1 per geographical area) with a mix of people with dementia, family carers and professionals. Each of the 3 groups met twice, to co‐design the intervention. A final meeting was held with all participants. Table 2 outlines phases of the EBCD process with the number and type of stakeholders involved in meetings.

**Table 2:** Phases of EBCD.

Phase of EBCD process	Stakeholders and time of activities
Systematic literature review and qualitative interviews with stakeholders, Thematic analysis, Evidence of existing medicines self‐management interventions, and theoretical underpinnings, Create film and evidence summary	Research team. Systematic review conducted prior to funded study commencing, Interviews and video created at project phase one
1a. Priority setting	14 people with dementia and 6 family carers *3 meetings, all in person in a research institute based on hospital grounds,* January–February 2024
1b. Priority setting	5 health and social care professionals, *2 meetings, all online,* January‐February 2024
1c. Priority setting	Total 22. 6 people with dementia, 9 family carers, 7 health and social care professionals *1 meeting, all in person in a local community centre* February 2024
2a. Co‐designing the intervention	First co‐design meetings: Group 1: 7, Group 2: 10 Group 3: 9 *One professional attended online, all else in person in a research institute and/or based on hospital grounds,* Second co‐design meetings: Group 1: 9 Group 2: 10 Group 3:10, *All in person in a research institute and/or based on hospital grounds,* March‐April 2024
2b. Co‐designing the intervention	*All in person in a local community centre,* June 2024

## 5 Priority‐Setting Meetings

Current evidence from project phase one, including the film of people with mild‐moderate dementia with and without carers describing experiences of self‐managing medicines, and findings from our systematic review [14] were presented in 1a. Priority setting meetings with 14 people with dementia and 6 family carers, and 1b. Priority setting meetings with 5 health and social care professionals, enabled stakeholders to discuss and reflect on key issues related to medicines‐self management with people living with dementia and family carers.

Five key priorities were identified from the 1a and 1b priority‐setting meetings:

1. How to get most out of services available
2. Knowing your medicines
3. Planning for the future
4. People who can help me
5. Making it right for me.

### 5.1 Joint Priority‐Setting Meeting

The five key priorities identified were not reduced to three as intended at the joint priority‐setting event. It was decided that all priorities were important for different people according to their fluctuating needs and the support available. At the joint priority setting meeting, the group agreed that the intervention should therefore be personalisable; the key priority areas should be brought together in one guide driven by conversations with the professional delivering the guide, where the person could pick and choose what their priority area or areas should be. The guide therefore needed to be designed in such a way that, with professional support, the person could easily and quickly navigate its contents.

## 6 Co‐Designing the Intervention Meetings

With a focus on making the guide personalisable, in the co‐design meetings, stakeholders discussed the priorities in the context of who, in detail, would be using the intervention. Stakeholders then discussed what the person living with dementia and family carer could think, feel, and do, because of the intervention; and therefore, what they should know, and how the intervention should be designed.

A protype of the intervention was created by the research team, patient public involvement group and expert facilitator by refining the content from 2a. The content was formatted into a guide for the person living with dementia and family carers to use, delivered by a professional who worked in the community with people with mild‐moderate dementia.

A celebration event was held following the six co‐design meetings. The event was an opportunity to gain feedback on the prototype of the guide but was a celebration with stakeholders giving short speeches about the social impact of the process and the developing intervention. Stakeholders were given a presentation by the research team, summarising the initial research evidence including the systematic literature review and project phase 1; and process and outcomes of the priority setting and co‐design meetings. Stakeholders discussed a sample of the prototype in three different designs and asked to discuss the content and design in groups. Feedback was particularly focused on the design of the guide, such as accessible font, use of photos as opposed to infographics.

## 7 Feedback From Stakeholders

In the final 2b meeting we sought feedback on all phases of the process from stakeholders. Important social impacts for the stakeholders were identified, such as support in their own personal journeys, feeling empowered to speak, hearing others' views from different cultural backgrounds, and sources of support. Stakeholders felt the process was successful in co‐creating an intervention based on real lived experiences. They also felt it gave them an opportunity to give something back. Some areas of improvement included the need for the film to be shown at later phases of the process, and issues with acoustics.

## 8 Refining the Intervention

The research team and patient public involvement group continued to refine the content and design of the guide prototype. The external facilitator created new designs based on this and the feedback from 2b. A video of the guide was also developed as suggested in the final celebration event. Having a verbal account of the guide was thought to increase accessibility, particularly for those whom English is not their first language. For some it may be easier to understand spoken English than written English. The patient public involvement group identified from the stakeholder feedback that a reminder of goals and actions could also be created, and therefore a postcard of goals and actions was developed that could be sent to the participant mid‐intervention.

Thus, the final content was brought together in a guide for the person with dementia and family carers. The guide encourages the person to identify key goals and actions to self‐manage their medicines based on at least one of the key priority areas designed to support active medicines self‐management underpinned by social cognitive, self‐efficacy [16, 17] and resilience [18] theories. For example, demonstrations of medicines self‐management strategies from interviewees were incorporated into the guide to enhance knowledge and skills. In the ‘Making it right for you’ section of the guide there is an example of moving medicines to where they are taken at night to facilitate adherence, based on real experiences of patients propping up safe medicine management systems. The sections of the guide are as follows:

– ‘Welcome page’. A key section designed to help the person with dementia and family carer think about what sections of the guide would be helpful for them. The guide was designed to be flexible to the needs and preferences of the individual, with the person able to quickly move to relevant topics rather than move through each section of the guide sequentially.
– ‘How can living with dementia affect how you organise medicines?’ encouraged discussion around what living with dementia is like for the person (and their family carer if relevant), and the implications it has for how they organise medicines.
– ‘Knowing your medicines’ helps them to consider what questions or problems they may have regarding their medicines. The guide does not cover information on specific medicines. Instead, it clarifies where someone may be able to access this information.
– ‘Who can help?’ provides information on which professionals can help and how.
– ‘Making it right for you’ considers the different aspects of medicines self‐management, identifying the current level of skills and knowledge with the person and what is required.
– ‘Planning for the future’ ways of planning, and the potential emotional challenges related to this.
– ‘All of my goals and actions’

A video of the guide for the person living with dementia and family carers was developed. The video gives a brief overview of sections of the guide and emphasises the need to identify clear goals and actions related to medicines self‐management.

A reminder postcard for the person with dementia and family carers was created. The postcard has a space for a reminder of individualised key goals and actions relevant to be sent to the person.

The professional delivering the intervention is an integral part of the intervention, helping the person decide what is relevant to them, and identify the key goals and actions. Given the emphasis on the intervention needing to be a facilitated conversation between the professional, person and family member; the research team also created a guide for professionals to support delivery of the intervention, as well as training. The professional revisits the person in the follow‐up visit 1 month later, either remotely or face to face, to provide any further support the person needs to take appropriate actions and achieve their goals.

Implementation materials included:

- A guide for professionals to deliver the intervention. This included an outline of seven key steps for the professional to follow as outlined in Figure 1.
- Training for professionals to deliver the intervention. Two sessions were developed by the research team to be delivered by three members of the research team.

**Figure 1:**
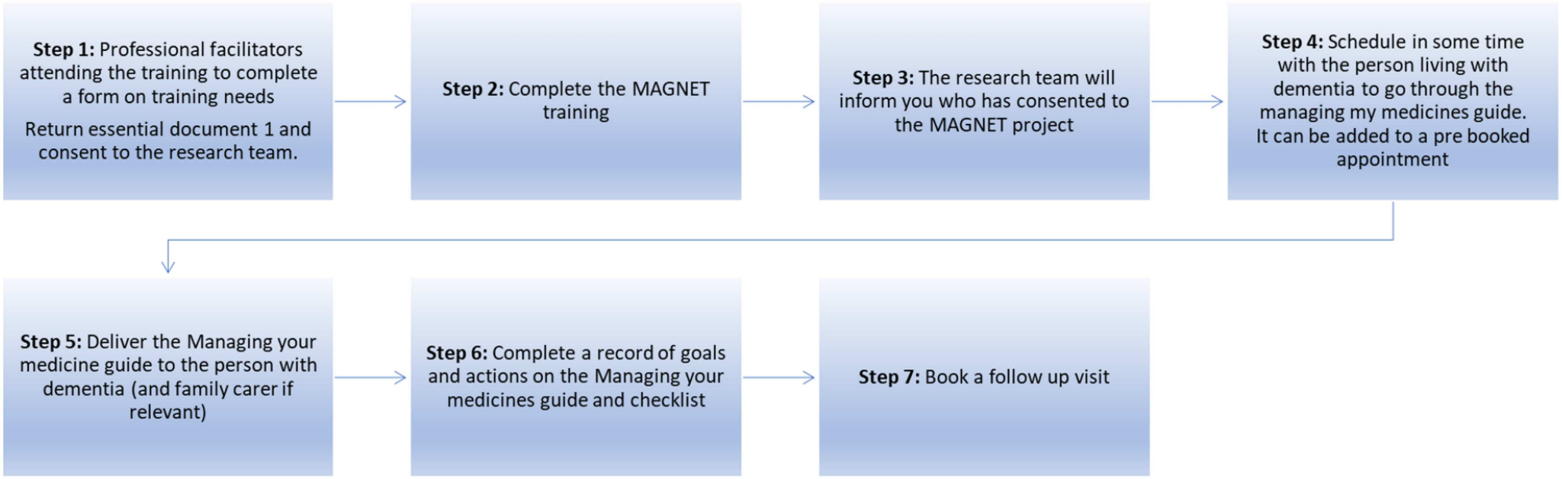
Key steps for professionals.

A logic model was developed by the research team to underpin the intervention, illustrated in Figure 2. The model was drawn from existing research evidence, such as our systematic review [14], preliminary empirical evidence from earlier project phases, key theories such as resilience theory [18], cognitive and self‐efficacy theories [25, 29] and the consolidated framework for implementation research [30], and learning from the co‐design meetings.

**Figure 2:**
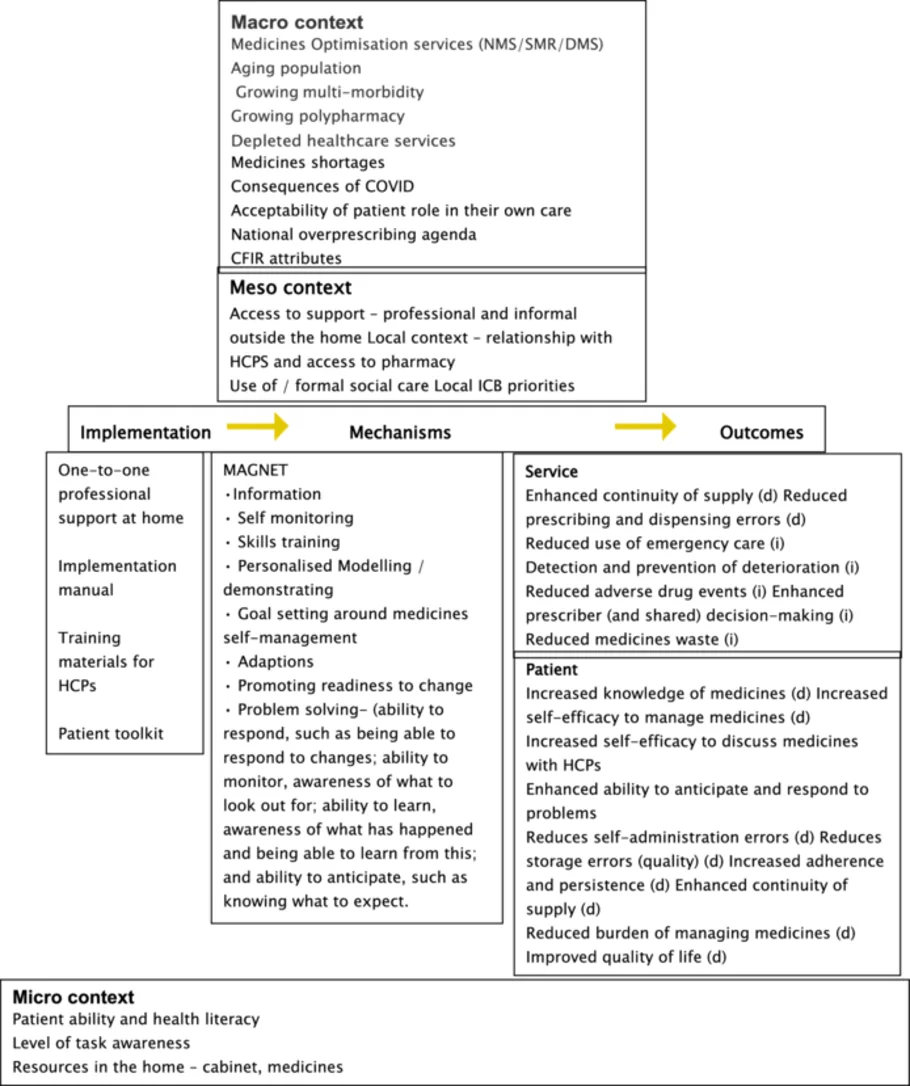
Logic model. CFIR, consolidated framework for implementation research; d, direct; DMS, discharge medicines service; HCP, health care professionals; i, indirect; ICB, Integrated Care Board; NMS, new medicines service; SMR, structured medication review.

## 9 Discussion

This is the first known study to co‐design a psychosocial intervention to empower community dwelling people with mild‐moderate dementia with and without family carers, to self‐manage their medicines, underpinned by social cognitive, self‐efficacy [25, 26] and resilient healthcare [18] theory. Five key priorities were identified. These were ‘how can living with dementia affect how you organise medicines’, ‘knowing my medicines’, ‘who can help’, ‘making it right for you’ and ‘planning for the future’. The intervention, supporting logic model and implementation materials were developed to address these priorities in the form of a guide, video and reminder postcard for the person, and training and an implementation guide for professionals. The feasibility and acceptability of this co‐designed community‐based psychosocial intervention has been tested in project phase 3 and will be reported elsewhere.

An intervention aiming for a holistic person‐centred understanding of medicines self‐management was created. The co‐design approach was critical in enabling us to develop an intervention that addressed real lived experiences of medicines self‐management among people living with dementia, family carers and professionals. The intervention goes beyond focusing only on adherence to medicine regimens, which previous interventions without co‐design have more often focused on [14], and addresses the broader experience of person's knowledge and understanding, supply management, monitoring effects and side effects and communicating with healthcare professionals, and addressing resilience capabilities. In dementia care, a person‐centred approach is important as social stigmas may lead to assumptions that people living with dementia are unable to take part in decisions about their own health [31, 32]. This co‐designed intervention development process enabled us to apply the evidence from project phase one where we elucidated how people with mild‐to‐moderate dementia, family carers and professionals are organising medicines, and through priority setting consider how this involvement in the medicines management system could be better supported, and develop an intervention that aims to provide this support through co‐design workshops.

A social impact of the co‐design process was evident through the creation of a supportive networking opportunity. Feedback from people with dementia and family carers was that the co‐design process had provided an important opportunity for expressing themselves on the experience of self‐managing medicines and finding tangible sources of support they were not aware of in the local area. This perhaps reflects the lack of community‐based medicines support following on from a dementia diagnosis [7]. Moreover, empowerment and focus on giving back to the community were benefits in the process. People living with dementia, and their carers are seldom heard voices in research, who took part in this process. The co‐design process had in a sense been an intervention for a local community of stakeholders. Some of the positive social impacts in a group of people living with dementia, family carers and professionals in this study, have been identified in other EBCD projects, such as feeling responsible and making a difference, supporting that it may be the EBCD method itself which helps enable these social impacts [20].

There were limitations to the co‐design process. Stakeholders fed back that they had forgotten about the film later in the co‐design process. For this population, showing the film more frequently at different meetings may have facilitated the process by drawing stakeholders back to the original experiences people with dementia and their family carers faced in self‐managing their medicines. There were different facilitators across the meetings, this may potentially have a positive or negative impact on intervention development. For example, their knowledge and experience with people with dementia differed as one facilitator had long‐standing involvement in the project whereas the external facilitator was only involved for this project two phase. However, both facilitators were briefed by members of the research team about how to deliver the sessions. Moreover, one of the challenges was ensuring the balance between the research findings, and the perspectives of stakeholders. The EBCD process, does require a time commitment of several months. From the first meeting to the final intervention and implementation materials took several months, and there was additional time spent in organising the several meetings, bringing multiple stakeholders together, and ensuring people who were from different cultural backgrounds, were supported and have equal power. Power dynamics are frequently cited as a challenge in EBCD, yet the process itself can help to support equal power dynamics through holding workshops, small co‐design teams which are delivered by trained facilitators [20]. Feedback indicated that stakeholders felt equally heard. However, the time spent in the process itself is also a strength in that the process enables stakeholders and the research team to have time and space to reflect on what is needed in the intervention and amend accordingly.

## 10 Conclusion

Our intervention takes a person‐centred approach to supporting medicines self‐management for people living with dementia and family carers. The intervention has the potential to enhance patient outcomes such as increasing knowledge and self‐efficacy to self‐manage medicines; and service outcomes such as detection and prevention of deterioration and reduce use of emergency care. Reflecting the EBCD process, we have shown how often underrepresented people with dementia, family carers and professionals involved in dementia care in the community, can co‐create an intervention which aims to enhance the medicines management system.

## Author Contributions

**Catherine Powell:** conceptualization, investigation, funding acquisition, writing – original draft, methodology, validation, visualization, writing – review and editing, formal analysis, project administration, data curation, supervision, resources. **Lubena Mirza:** writing – review and editing, conceptualization, methodology, investigation, formal analysis, project administration. **Mohammed Akhlak Rauf:** funding acquisition, conceptualization, investigation, methodology, writing – review and editing, formal analysis. **Justine Tomlinson:** funding acquisition, conceptualization, investigation, methodology, writing – review and editing, formal analysis. **Zoe Edwards:** funding acquisition, conceptualization, investigation, methodology, writing – review and editing, formal analysis. **Rozeena Khan:** writing – review and editing, formal analysis, methodology. **Jan Speechley:** writing – review and editing, formal analysis, methodology. **John L. O'Dwyer:** funding acquisition, conceptualization, investigation, methodology, formal analysis. **Catherine Quinn:** funding acquisition, formal analysis, conceptualization, investigation, methodology. **Beth Fylan:** conceptualization, investigation, funding acquisition, methodology, writing – review and editing, formal analysis, supervision.

## Ethics Statement

Ethical approval was granted by the Ethics committee of Health Research Authority REC: 23/PR/0180 IRAS: 320482.

## Conflicts of Interest

The authors declare no conflict of interest.

## Supporting information


Supporting File

## Data Availability

Data sharing not applicable to this article as no datasets were generated or analysed during the current study.
